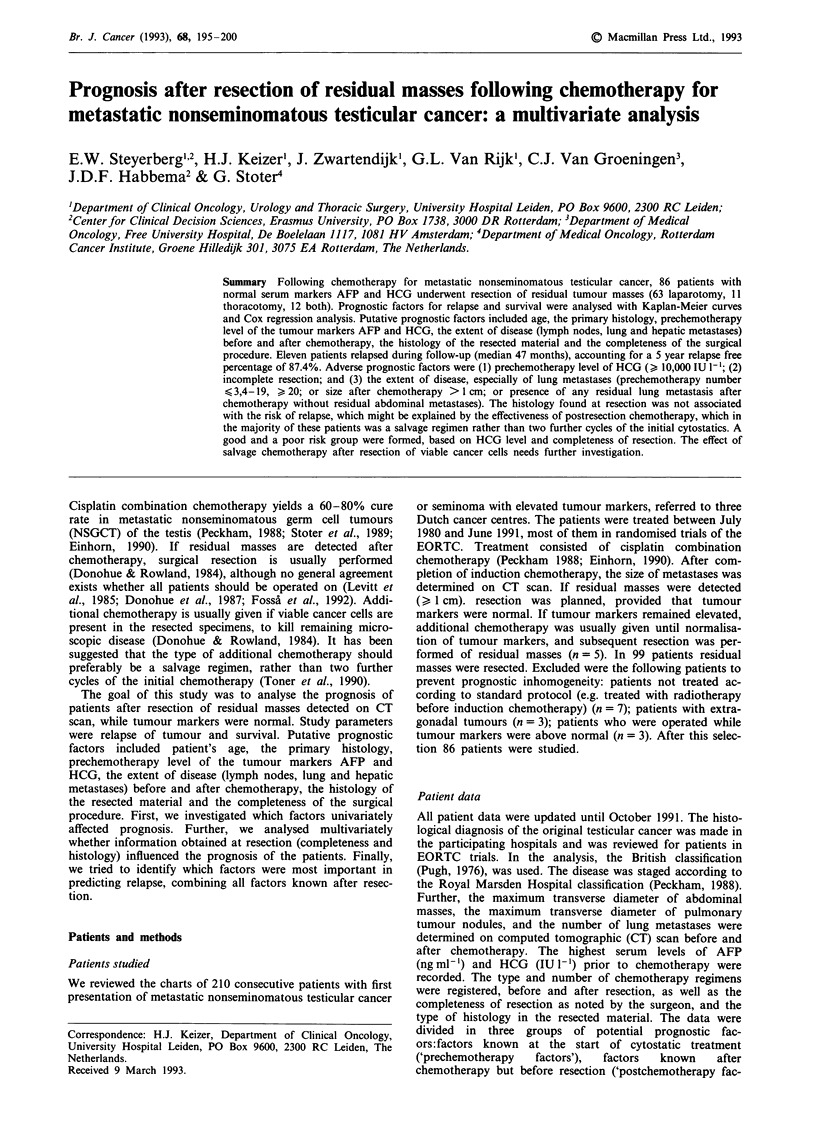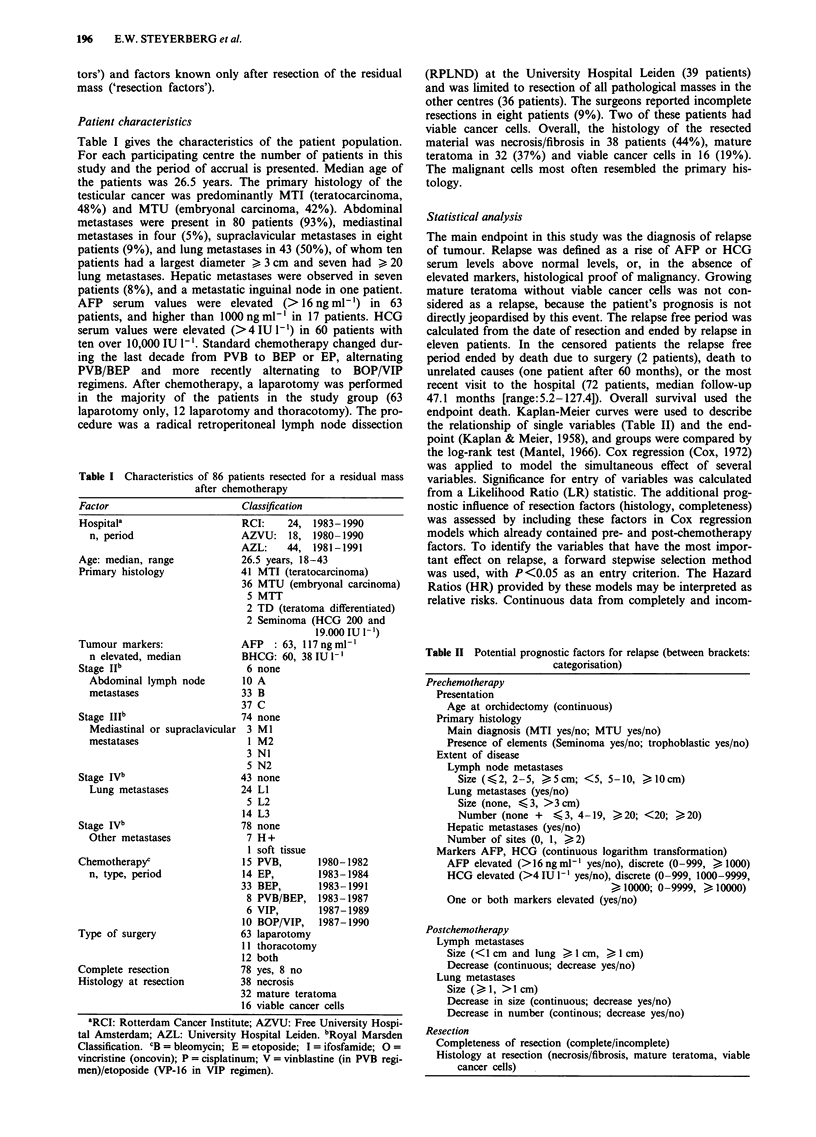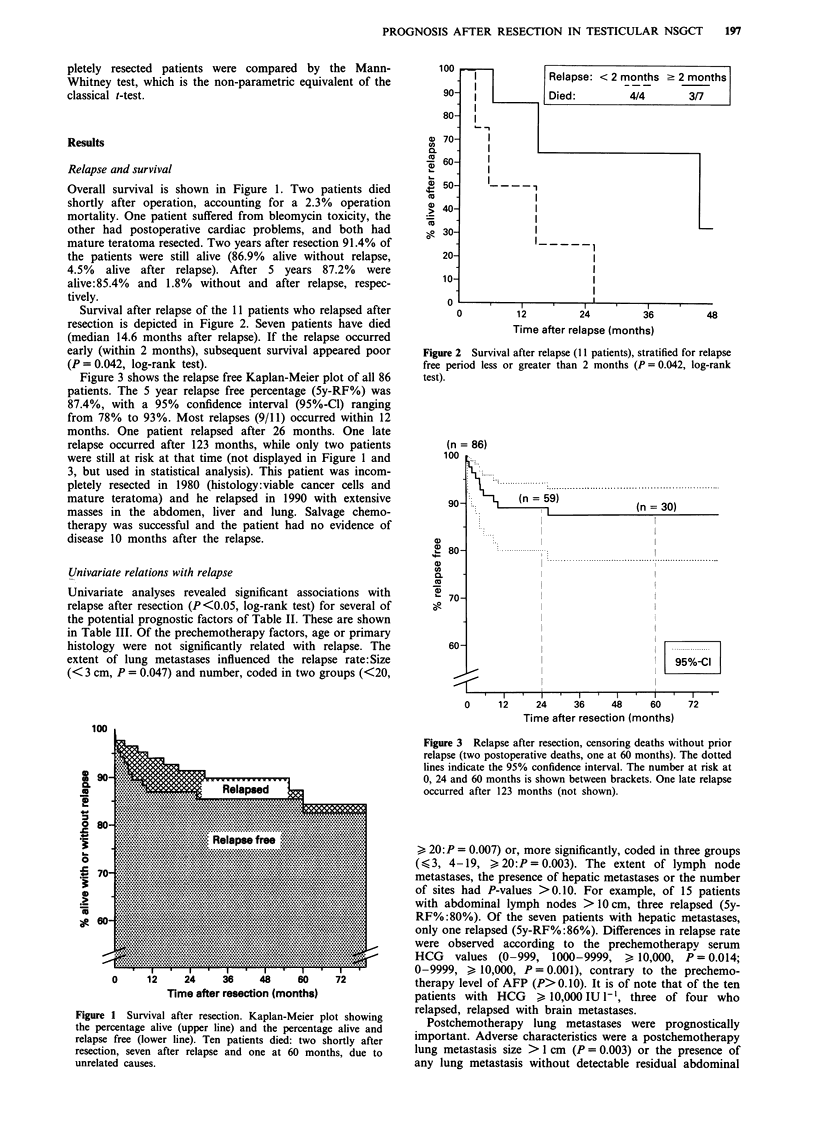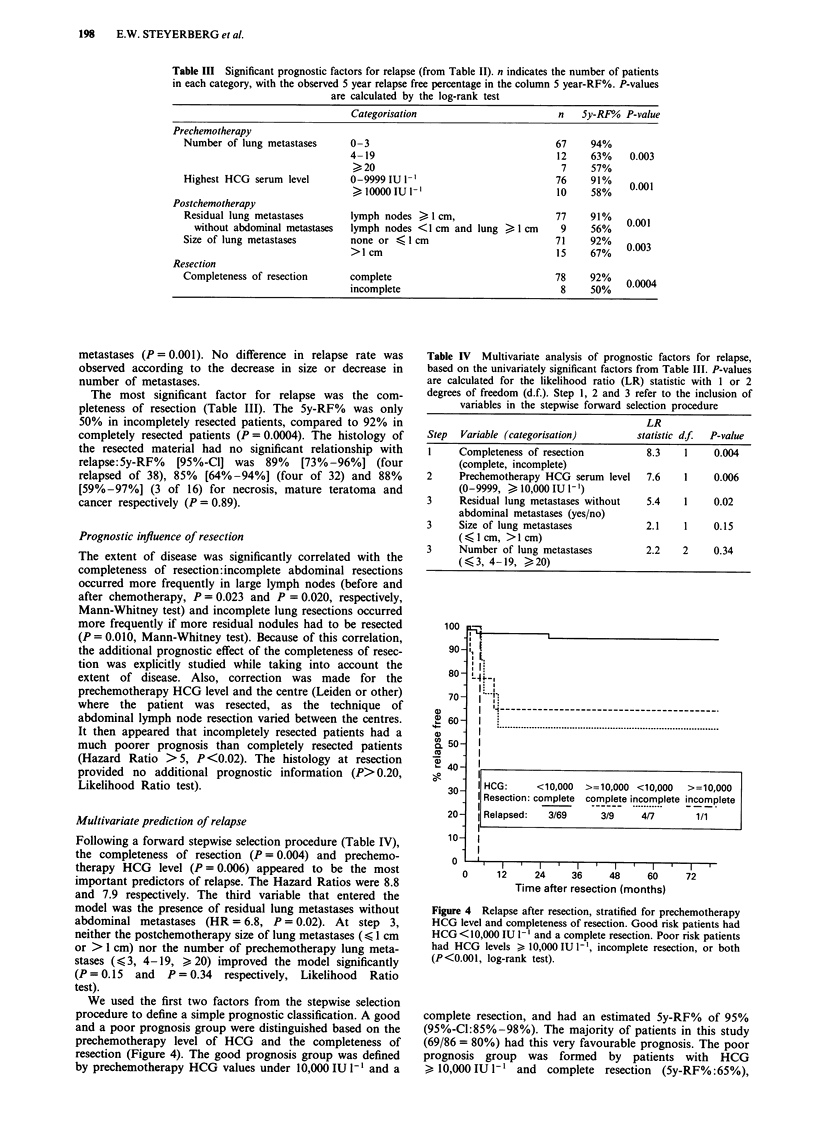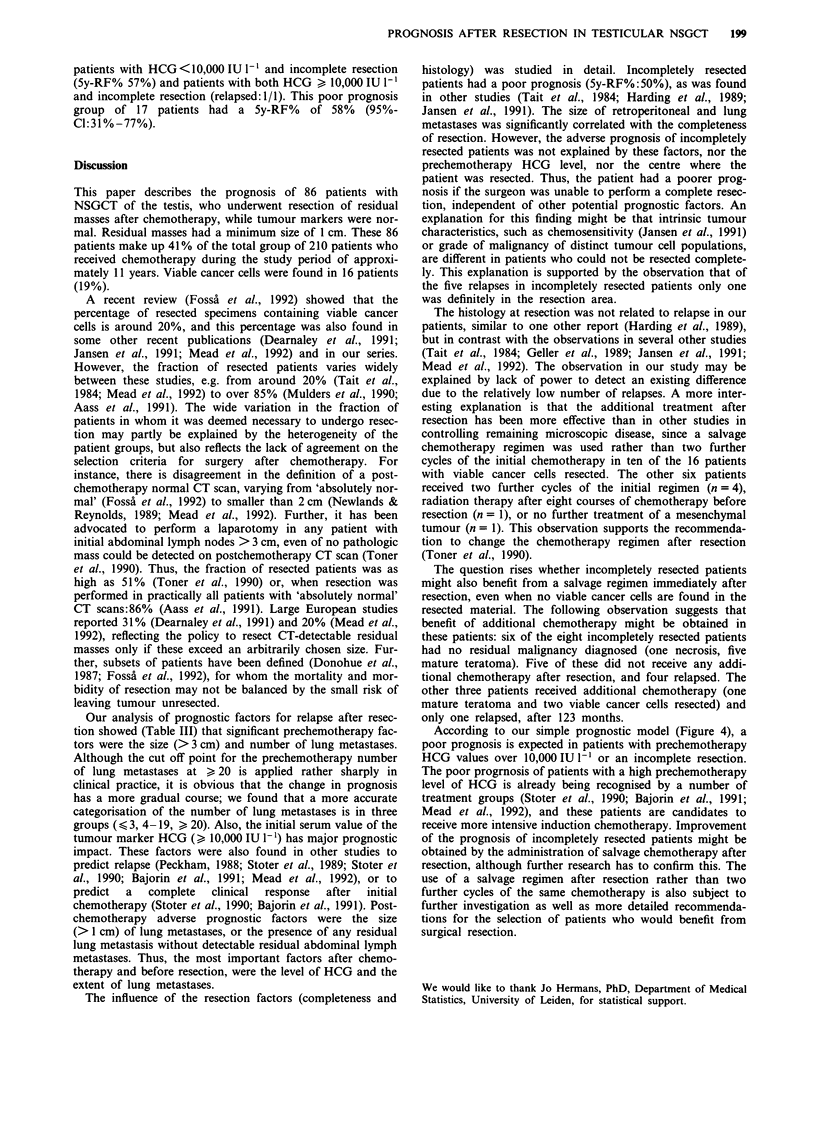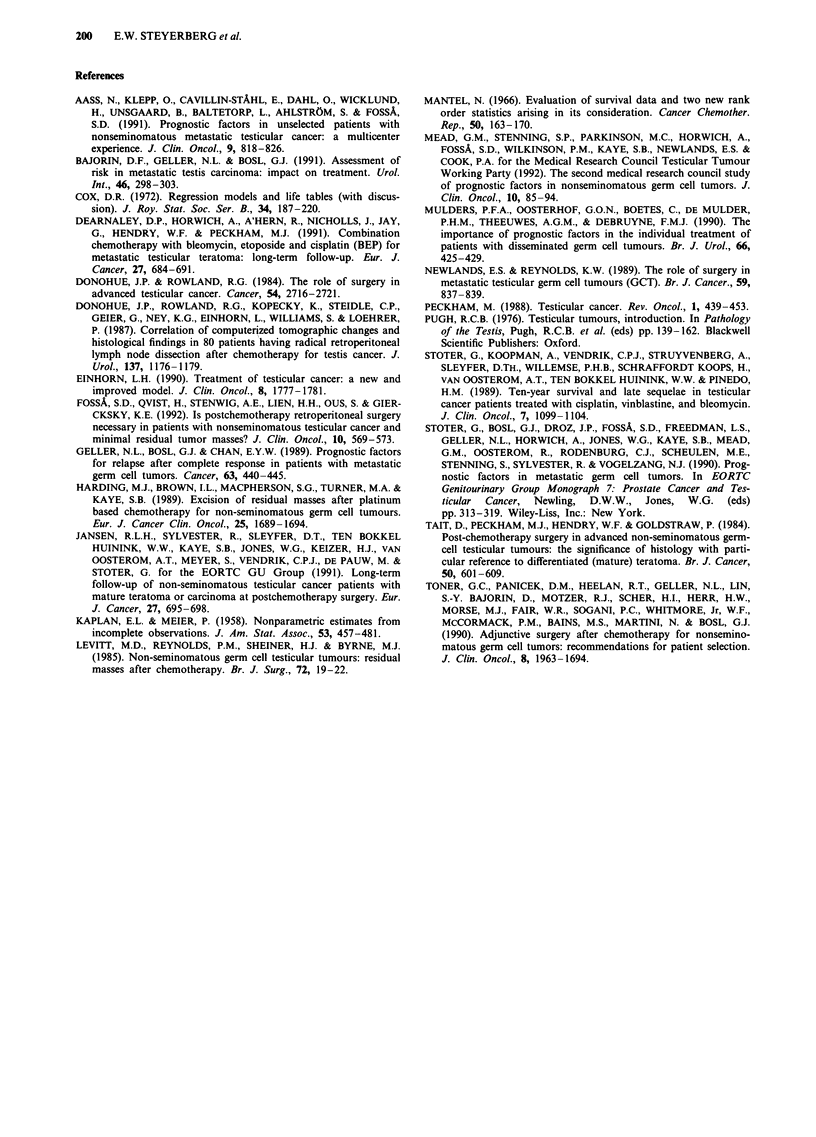# Prognosis after resection of residual masses following chemotherapy for metastatic nonseminomatous testicular cancer: a multivariate analysis.

**DOI:** 10.1038/bjc.1993.313

**Published:** 1993-07

**Authors:** E. W. Steyerberg, H. J. Keizer, J. Zwartendijk, G. L. Van Rijk, C. J. Van Groeningen, J. D. Habbema, G. Stoter

**Affiliations:** Department of Clinical Oncology, Urology and Thoracic Surgery, University Hospital Leiden, The Netherlands.

## Abstract

Following chemotherapy for metastatic nonseminomatous testicular cancer, 86 patients with normal serum markers AFP and HCG underwent resection of residual tumour masses (63 laparotomy, 11 thoracotomy, 12 both). Prognostic factors for relapse and survival were analysed with Kaplan-Meier curves and Cox regression analysis. Putative prognostic factors included age, the primary histology, prechemotherapy level of the tumour markers AFP and HCG, the extent of disease (lymph nodes, lung and hepatic metastases) before and after chemotherapy, the histology of the resected material and the completeness of the surgical procedure. Eleven patients relapsed during follow-up (median 47 months), accounting for a 5 year relapse free percentage of 87.4%. Adverse prognostic factors were (1) prechemotherapy level of HCG (> or = 10,000 IU l-1; (2) incomplete resection; and (3) the extent of disease, especially of lung metastases (prechemotherapy number < or = 3,4-19, > or = 20; or size after chemotherapy > 1 cm; or presence of any residual lung metastasis after chemotherapy without residual abdominal metastases). The histology found at resection was not associated with the risk of relapse, which might be explained by the effectiveness of postresection chemotherapy, which in the majority of these patients was a salvage regimen rather than two further cycles of the initial cytostatics. A good and a poor risk group were formed, based on HCG level and completeness of resection. The effect of salvage chemotherapy after resection of viable cancer cells needs further investigation.


Br. J. Cancer (1993), 68, 195–200 © Macmillan Press Ltd., 1993

### Prognosis after resection of residual masses following chemotherapy for metastatic nonseminomatous testicular cancer: a multivariate analysis

E.W. Steyerberg[1,2], H.J. Keizer[1], J. Zwartendijk[1], G.L. Van Rijk[1], C.J. Van Groeningen[3], J.D.F. Habbema[2] & G. Stoter[4]

[1]*Department of Clinical Oncology, Urology and Thoracic Surgery, University Hospital Leiden, PO Box 9600, 2300 RC Leiden;* [2]*Center for Clinical Decision Sciences, Erasmus University, PO Box 1738, 3000 DR Rotterdam;* [3]*Department of Medical Oncology, Free University Hospital, De Boelelaan 1117, 1081 HV Amsterdam;* [4]*Department of Medical Oncology, Rotterdam Cancer Institute, Groene Hilledijk 301, 3075 EA Rotterdam, The Netherlands.*

**Summary** Following chemotherapy for metastatic nonseminomatous testicular cancer, 86 patients with normal serum markers AFP and HCG underwent resection of residual tumour masses (63 laparotomy, 11 thoracotomy, 12 both). Prognostic factors for relapse and survival were analysed with Kaplan-Meier curves and Cox regression analysis. Putative prognostic factors included age, the primary histology, prechemotherapy level of the tumour markers AFP and HCG, the extent of disease (lymph nodes, lung and hepatic metastases) before and after chemotherapy, the histology of the resected material and the completeness of the surgical procedure. Eleven patients relapsed during follow-up (median 47 months), accounting for a 5 year relapse free percentage of 87.4%. Adverse prognostic factors were (1) prechemotherapy level of HCG ($\geqslant$ 10,000 IU $l^{-1}$; (2) incomplete resection; and (3) the extent of disease, especially of lung metastases (prechemotherapy number $\leqslant$3,4–19, $\geqslant$20; or size after chemotherapy $>$1 cm; or presence of any residual lung metastasis after chemotherapy without residual abdominal metastases). The histology found at resection was not associated with the risk of relapse, which might be explained by the effectiveness of postresection chemotherapy, which in the majority of these patients was a salvage regimen rather than two further cycles of the initial cytostatics. A good and a poor risk group were formed, based on HCG level and completeness of resection. The effect of salvage chemotherapy after resection of viable cancer cells needs further investigation.

Cisplatin combination chemotherapy yields a 60–80% cure rate in metastatic nonseminomatous germ cell tumours (NSGCT) of the testis (Peckham, 1988; Stoter *et al.*, 1989; Einhorn, 1990). If residual masses are detected after chemotherapy, surgical resection is usually performed (Donohue & Rowland, 1984), although no general agreement exists whether all patients should be operated on (Levitt *et al.*, 1985; Donohue *et al.*, 1987; Fosså *et al.*, 1992). Additional chemotherapy is usually given if viable cancer cells are present in the resected specimens, to kill remaining microscopic disease (Donohue & Rowland, 1984). It has been suggested that the type of additional chemotherapy should preferably be a salvage regimen, rather than two further cycles of the initial chemotherapy (Toner *et al.*, 1990).

The goal of this study was to analyse the prognosis of patients after resection of residual masses detected on CT scan, while tumour markers were normal. Study parameters were relapse of tumour and survival. Putative prognostic factors included patient's age, the primary histology, prechemotherapy level of the tumour markers AFP and HCG, the extent of disease (lymph nodes, lung and hepatic metastases) before and after chemotherapy, the histology of the resected material and the completeness of the surgical procedure. First, we investigated which factors univariately affected prognosis. Further, we analysed multivariately whether information obtained at resection (completeness and histology) influenced the prognosis of the patients. Finally, we tried to identify which factors were most important in predicting relapse, combining all factors known after resection.

### Patients and methods

### *Patients studied*

We reviewed the charts of 210 consecutive patients with first presentation of metastatic nonseminomatous testicular cancer or seminoma with elevated tumour markers, referred to three Dutch cancer centres. The patients were treated between July 1980 and June 1991, most of them in randomised trials of the EORTC. Treatment consisted of cisplatin combination chemotherapy (Peckham 1988; Einhorn, 1990). After completion of induction chemotherapy, the size of metastases was determined on CT scan. If residual masses were detected ($\geqslant$ 1 cm). resection was planned, provided that tumour markers were normal. If tumour markers remained elevated, additional chemotherapy was usually given until normalisation of tumour markers, and subsequent resection was performed of residual masses ($n = 5$). In 99 patients residual masses were resected. Excluded were the following patients to prevent prognostic inhomogeneity: patients not treated according to standard protocol (e.g. treated with radiotherapy before induction chemotherapy) ($n = 7$); patients with extragonadal tumours ($n = 3$); patients who were operated while tumour markers were above normal ($n = 3$). After this selection 86 patients were studied.

Correspondence: H.J. Keizer, Department of Clinical Oncology, University Hospital Leiden, PO Box 9600, 2300 RC Leiden, The Netherlands.
Received 9 March 1993.

### *Patient data*

All patient data were updated until October 1991. The histological diagnosis of the original testicular cancer was made in the participating hospitals and was reviewed for patients in EORTC trials. In the analysis, the British classification (Pugh, 1976), was used. The disease was staged according to the Royal Marsden Hospital classification (Peckham, 1988). Further, the maximum transverse diameter of abdominal masses, the maximum transverse diameter of pulmonary tumour nodules, and the number of lung metastases were determined on computed tomographic (CT) scan before and after chemotherapy. The highest serum levels of AFP (ng $ml^{-1}$) and HCG (IU $l^{-1}$) prior to chemotherapy were recorded. The type and number of chemotherapy regimens were registered, before and after resection, as well as the completeness of resection as noted by the surgeon, and the type of histology in the resected material. The data were divided in three groups of potential prognostic factors:factors known at the start of cytostatic treatment ('prechemotherapy factors'), factors known after chemotherapy but before resection ('postchemotherapy factors') and factors known only after resection of the residual mass ('resection factors').

*Patient characteristics*

Table I gives the characteristics of the patient population. For each participating centre the number of patients in this study and the period of accrual is presented. Median age of the patients was 26.5 years. The primary histology of the testicular cancer was predominantly MTI (teratocarcinoma, 48%) and MTU (embryonal carcinoma, 42%). Abdominal metastases were present in 80 patients (93%), mediastinal metastases in four (5%), supraclavicular metastases in eight patients (9%), and lung metastases in 43 (50%), of whom ten patients had a largest diameter $\geqslant 3$ cm and seven had $\geqslant 20$ lung metastases. Hepatic metastases were observed in seven patients (8%), and a metastatic inguinal node in one patient. AFP serum values were elevated ($>16$ ng ml$^{-1}$) in 63 patients, and higher than 1000 ng ml$^{-1}$ in 17 patients. HCG serum values were elevated ($>4$ IU l$^{-1}$) in 60 patients with ten over 10,000 IU l$^{-1}$. Standard chemotherapy changed during the last decade from PVB to BEP or EP, alternating PVB/BEP and more recently alternating to BOP/VIP regimens. After chemotherapy, a laparotomy was performed in the majority of the patients in the study group (63 laparotomy only, 12 laparotomy and thoracotomy). The procedure was a radical retroperitoneal lymph node dissection (RPLND) at the University Hospital Leiden (39 patients) and was limited to resection of all pathological masses in the other centres (36 patients). The surgeons reported incomplete resections in eight patients (9%). Two of these patients had viable cancer cells. Overall, the histology of the resected material was necrosis/fibrosis in 38 patients (44%), mature teratoma in 32 (37%) and viable cancer cells in 16 (19%). The malignant cells most often resembled the primary histology.

**Table I** Characteristics of 86 patients resected for a residual mass after chemotherapy

| *Factor* | *Classification* |
|---|---|
| Hospital[a] | RCI: 24, 1983–1990 |
| n, period | AZVU: 18, 1980–1990 |
| | AZL: 44, 1981–1991 |
| Age: median, range | 26.5 years, 18–43 |
| Primary histology | 41 MTI (teratocarcinoma) |
| | 36 MTU (embryonal carcinoma) |
| | 5 MTT |
| | 2 TD (teratoma differentiated) |
| | 2 Seminoma (HCG 200 and 19.000 IU l$^{-1}$) |
| Tumour markers: | AFP : 63, 117 ng ml$^{-1}$ |
| n elevated, median | BHCG: 60, 38 IU l$^{-1}$ |
| Stage II[b] | 6 none |
| Abdominal lymph node | 10 A |
| metastases | 33 B |
| | 37 C |
| Stage III[b] | 74 none |
| Mediastinal or supraclavicular | 3 M1 |
| mestatases | 1 M2 |
| | 3 N1 |
| | 5 N2 |
| Stage IV[b] | 43 none |
| Lung metastases | 24 L1 |
| | 5 L2 |
| | 14 L3 |
| Stage IV[b] | 78 none |
| Other metastases | 7 H+ |
| | 1 soft tissue |
| Chemotherapy[c] | 15 PVB, 1980–1982 |
| n, type, period | 14 EP, 1983–1984 |
| | 33 BEP, 1983–1991 |
| | 8 PVB/BEP, 1983–1987 |
| | 6 VIP, 1987–1989 |
| | 10 BOP/VIP, 1987–1990 |
| Type of surgery | 63 laparotomy |
| | 11 thoracotomy |
| | 12 both |
| Complete resection | 78 yes, 8 no |
| Histology at resection | 38 necrosis |
| | 32 mature teratoma |
| | 16 viable cancer cells |

[a]RCI: Rotterdam Cancer Institute; AZVU: Free University Hospital Amsterdam; AZL: University Hospital Leiden. [b]Royal Marsden Classification. [c]B = bleomycin; E = etoposide; I = ifosfamide; O = vincristine (oncovin); P = cisplatinum; V = vinblastine (in PVB regimen)/etoposide (VP-16 in VIP regimen).

*Statistical analysis*

The main endpoint in this study was the diagnosis of relapse of tumour. Relapse was defined as a rise of AFP or HCG serum levels above normal levels, or, in the absence of elevated markers, histological proof of malignancy. Growing mature teratoma without viable cancer cells was not considered as a relapse, because the patient's prognosis is not directly jeopardised by this event. The relapse free period was calculated from the date of resection and ended by relapse in eleven patients. In the censored patients the relapse free period ended by death due to surgery (2 patients), death to unrelated causes (one patient after 60 months), or the most recent visit to the hospital (72 patients, median follow-up 47.1 months [range:5.2–127.4]). Overall survival used the endpoint death. Kaplan-Meier curves were used to describe the relationship of single variables (Table II) and the endpoint (Kaplan & Meier, 1958), and groups were compared by the log-rank test (Mantel, 1966). Cox regression (Cox, 1972) was applied to model the simultaneous effect of several variables. Significance for entry of variables was calculated from a Likelihood Ratio (LR) statistic. The additional prognostic influence of resection factors (histology, completeness) was assessed by including these factors in Cox regression models which already contained pre- and post-chemotherapy factors. To identify the variables that have the most important effect on relapse, a forward stepwise selection method was used, with $P<0.05$ as an entry criterion. The Hazard Ratios (HR) provided by these models may be interpreted as relative risks. Continuous data from completely and incom-

**Table II** Potential prognostic factors for relapse (between brackets: categorisation)

| |
|---|
| *Prechemotherapy* |
| Presentation |
| Age at orchidectomy (continuous) |
| Primary histology |
| Main diagnosis (MTI yes/no; MTU yes/no) |
| Presence of elements (Seminoma yes/no; trophoblastic yes/no) |
| Extent of disease |
| Lymph node metastases |
| Size ($\leqslant 2$, 2–5, $\geqslant 5$ cm; $<5$, 5–10, $\geqslant 10$ cm) |
| Lung metastases (yes/no) |
| Size (none, $\leqslant 3$, $>3$ cm) |
| Number (none + $\leqslant 3$, 4–19, $\geqslant 20$; $<20$; $\geqslant 20$) |
| Hepatic metastases (yes/no) |
| Number of sites (0, 1, $\geqslant 2$) |
| Markers AFP, HCG (continuous logarithm transformation) |
| AFP elevated ($>16$ ng ml$^{-1}$ yes/no), discrete (0–999, $\geqslant 1000$) |
| HCG elevated ($>4$ IU l$^{-1}$ yes/no), discrete (0–999, 1000–9999, $\geqslant 10000$; 0–9999, $\geqslant 10000$) |
| One or both markers elevated (yes/no) |
| *Postchemotherapy* |
| Lymph metastases |
| Size ($<1$ cm and lung $\geqslant 1$ cm, $\geqslant 1$ cm) |
| Decrease (continuous; decrease yes/no) |
| Lung metastases |
| Size ($\geqslant 1$, $>1$ cm) |
| Decrease in size (continuous; decrease yes/no) |
| Decrease in number (continous; decrease yes/no) |
| *Resection* |
| Completeness of resection (complete/incomplete) |
| Histology at resection (necrosis/fibrosis, mature teratoma, viable cancer cells) |

pletely resected patients were compared by the Mann-Whitney test, which is the non-parametric equivalent of the classical *t*-test.

### Results

### *Relapse and survival*

Overall survival is shown in Figure 1. Two patients died shortly after operation, accounting for a 2.3% operation mortality. One patient suffered from bleomycin toxicity, the other had postoperative cardiac problems, and both had mature teratoma resected. Two years after resection 91.4% of the patients were still alive (86.9% alive without relapse, 4.5% alive after relapse). After 5 years 87.2% were alive:85.4% and 1.8% without and after relapse, respectively.

Survival after relapse of the 11 patients who relapsed after resection is depicted in Figure 2. Seven patients have died (median 14.6 months after relapse). If the relapse occurred early (within 2 months), subsequent survival appeared poor ($P = 0.042$, log-rank test).

Figure 3 shows the relapse free Kaplan-Meier plot of all 86 patients. The 5 year relapse free percentage (5y-RF%) was 87.4%, with a 95% confidence interval (95%-Cl) ranging from 78% to 93%. Most relapses (9/11) occurred within 12 months. One patient relapsed after 26 months. One late relapse occurred after 123 months, while only two patients were still at risk at that time (not displayed in Figure 1 and 3, but used in statistical analysis). This patient was incompletely resected in 1980 (histology:viable cancer cells and mature teratoma) and he relapsed in 1990 with extensive masses in the abdomen, liver and lung. Salvage chemotherapy was successful and the patient had no evidence of disease 10 months after the relapse.

### *Univariate relations with relapse*

Univariate analyses revealed significant associations with relapse after resection ($P < 0.05$, log-rank test) for several of the potential prognostic factors of Table II. These are shown in Table III. Of the prechemotherapy factors, age or primary histology were not significantly related with relapse. The extent of lung metastases influenced the relapse rate:Size (<3 cm, $P = 0.047$) and number, coded in two groups (<20,

**Figure 1** Survival after resection. Kaplan-Meier plot showing the percentage alive (upper line) and the percentage alive and relapse free (lower line). Ten patients died: two shortly after resection, seven after relapse and one at 60 months, due to unrelated causes.

**Figure 2** Survival after relapse (11 patients), stratified for relapse free period less or greater than 2 months ($P = 0.042$, log-rank test).

**Figure 3** Relapse after resection, censoring deaths without prior relapse (two postoperative deaths, one at 60 months). The dotted lines indicate the 95% confidence interval. The number at risk at 0, 24 and 60 months is shown between brackets. One late relapse occurred after 123 months (not shown).

$\geqslant 20$:$P = 0.007$) or, more significantly, coded in three groups ($\leqslant 3$, 4–19, $\geqslant 20$:$P = 0.003$). The extent of lymph node metastases, the presence of hepatic metastases or the number of sites had *P*-values $>0.10$. For example, of 15 patients with abdominal lymph nodes $>10$ cm, three relapsed (5y-RF%:80%). Of the seven patients with hepatic metastases, only one relapsed (5y-RF%:86%). Differences in relapse rate were observed according to the prechemotherapy serum HCG values (0–999, 1000–9999, $\geqslant 10{,}000$, $P = 0.014$; 0–9999, $\geqslant 10{,}000$, $P = 0.001$), contrary to the prechemotherapy level of AFP ($P > 0.10$). It is of note that of the ten patients with HCG $\geqslant 10{,}000$ IU $l^{-1}$, three of four who relapsed, relapsed with brain metastases.

Postchemotherapy lung metastases were prognostically important. Adverse characteristics were a postchemotherapy lung metastasis size $>1$ cm ($P = 0.003$) or the presence of any lung metastasis without detectable residual abdominal

**Table III** Significant prognostic factors for relapse (from Table II). *n* indicates the number of patients in each category, with the observed 5 year relapse free percentage in the column 5 year-RF%. *P*-values are calculated by the log-rank test

| | *Categorisation* | *n* | *5y-RF%* | *P-value* |
|---|---|---|---|---|
| *Prechemotherapy* | | | | |
| Number of lung metastases | 0–3 | 67 | 94% | |
| | 4–19 | 12 | 63% | 0.003 |
| | ⩾20 | 7 | 57% | |
| Highest HCG serum level | 0–9999 IU $l^{-1}$ | 76 | 91% | 0.001 |
| | ⩾10000 IU $l^{-1}$ | 10 | 58% | |
| *Postchemotherapy* | | | | |
| Residual lung metastases without abdominal metastases | lymph nodes ⩾1 cm, | 77 | 91% | 0.001 |
| | lymph nodes <1 cm and lung ⩾1 cm | 9 | 56% | |
| Size of lung metastases | none or ⩽1 cm | 71 | 92% | 0.003 |
| | >1 cm | 15 | 67% | |
| *Resection* | | | | |
| Completeness of resection | complete | 78 | 92% | 0.0004 |
| | incomplete | 8 | 50% | |

metastases ($P = 0.001$). No difference in relapse rate was observed according to the decrease in size or decrease in number of metastases.

The most significant factor for relapse was the completeness of resection (Table III). The 5y-RF% was only 50% in incompletely resected patients, compared to 92% in completely resected patients ($P = 0.0004$). The histology of the resected material had no significant relationship with relapse:5y-RF% [95%-Cl] was 89% [73%–96%] (four relapsed of 38), 85% [64%–94%] (four of 32) and 88% [59%–97%] (3 of 16) for necrosis, mature teratoma and cancer respectively ($P = 0.89$).

### *Prognostic influence of resection*

The extent of disease was significantly correlated with the completeness of resection:incomplete abdominal resections occurred more frequently in large lymph nodes (before and after chemotherapy, $P = 0.023$ and $P = 0.020$, respectively, Mann-Whitney test) and incomplete lung resections occurred more frequently if more residual nodules had to be resected ($P = 0.010$, Mann-Whitney test). Because of this correlation, the additional prognostic effect of the completeness of resection was explicitly studied while taking into account the extent of disease. Also, correction was made for the prechemotherapy HCG level and the centre (Leiden or other) where the patient was resected, as the technique of abdominal lymph node resection varied between the centres. It then appeared that incompletely resected patients had a much poorer prognosis than completely resected patients (Hazard Ratio >5, $P < 0.02$). The histology at resection provided no additional prognostic information ($P > 0.20$, Likelihood Ratio test).

### *Multivariate prediction of relapse*

Following a forward stepwise selection procedure (Table IV), the completeness of resection ($P = 0.004$) and prechemotherapy HCG level ($P = 0.006$) appeared to be the most important predictors of relapse. The Hazard Ratios were 8.8 and 7.9 respectively. The third variable that entered the model was the presence of residual lung metastases without abdominal metastases (HR = 6.8, $P = 0.02$). At step 3, neither the postchemotherapy size of lung metastases (⩽1 cm or >1 cm) nor the number of prechemotherapy lung metastases (⩽3, 4–19, ⩾20) improved the model significantly ($P = 0.15$ and $P = 0.34$ respectively, Likelihood Ratio test).

We used the first two factors from the stepwise selection procedure to define a simple prognostic classification. A good and a poor prognosis group were distinguished based on the prechemotherapy level of HCG and the completeness of resection (Figure 4). The good prognosis group was defined by prechemotherapy HCG values under 10,000 IU $l^{-1}$ and a complete resection, and had an estimated 5y-RF% of 95% (95%-Cl:85%–98%). The majority of patients in this study (69/86 = 80%) had this very favourable prognosis. The poor prognosis group was formed by patients with HCG ⩾10,000 IU $l^{-1}$ and complete resection (5y-RF%:65%),

**Table IV** Multivariate analysis of prognostic factors for relapse, based on the univariately significant factors from Table III. *P*-values are calculated for the likelihood ratio (LR) statistic with 1 or 2 degrees of freedom (d.f.). Step 1, 2 and 3 refer to the inclusion of variables in the stepwise forward selection procedure

| *Step* | *Variable (categorisation)* | *LR statistic* | *d.f.* | *P-value* |
|---|---|---|---|---|
| 1 | Completeness of resection (complete, incomplete) | 8.3 | 1 | 0.004 |
| 2 | Prechemotherapy HCG serum level (0–9999, ⩾10,000 IU $l^{-1}$) | 7.6 | 1 | 0.006 |
| 3 | Residual lung metastases without abdominal metastases (yes/no) | 5.4 | 1 | 0.02 |
| 3 | Size of lung metastases (⩽1 cm, >1 cm) | 2.1 | 1 | 0.15 |
| 3 | Number of lung metastases (⩽3, 4–19, ⩾20) | 2.2 | 2 | 0.34 |

**Figure 4** Relapse after resection, stratified for prechemotherapy HCG level and completeness of resection. Good risk patients had HCG <10,000 IU $l^{-1}$ and a complete resection. Poor risk patients had HCG levels ⩾10,000 IU $l^{-1}$, incomplete resection, or both ($P < 0.001$, log-rank test).

patients with HCG < 10,000 IU $l^{-1}$ and incomplete resection (5y-RF% 57%) and patients with both HCG ⩾ 10,000 IU $l^{-1}$ and incomplete resection (relapsed: 1/1). This poor prognosis group of 17 patients had a 5y-RF% of 58% (95%-Cl: 31%–77%).

### Discussion

This paper describes the prognosis of 86 patients with NSGCT of the testis, who underwent resection of residual masses after chemotherapy, while tumour markers were normal. Residual masses had a minimum size of 1 cm. These 86 patients make up 41% of the total group of 210 patients who received chemotherapy during the study period of approximately 11 years. Viable cancer cells were found in 16 patients (19%).

A recent review (Fosså *et al.*, 1992) showed that the percentage of resected specimens containing viable cancer cells is around 20%, and this percentage was also found in some other recent publications (Dearnaley *et al.*, 1991; Jansen *et al.*, 1991; Mead *et al.*, 1992) and in our series. However, the fraction of resected patients varies widely between these studies, e.g. from around 20% (Tait *et al.*, 1984; Mead *et al.*, 1992) to over 85% (Mulders *et al.*, 1990; Aass *et al.*, 1991). The wide variation in the fraction of patients in whom it was deemed necessary to undergo resection may partly be explained by the heterogeneity of the patient groups, but also reflects the lack of agreement on the selection criteria for surgery after chemotherapy. For instance, there is disagreement in the definition of a post-chemotherapy normal CT scan, varying from 'absolutely normal' (Fosså *et al.*, 1992) to smaller than 2 cm (Newlands & Reynolds, 1989; Mead *et al.*, 1992). Further, it has been advocated to perform a laparotomy in any patient with initial abdominal lymph nodes > 3 cm, even of no pathologic mass could be detected on postchemotherapy CT scan (Toner *et al.*, 1990). Thus, the fraction of resected patients was as high as 51% (Toner *et al.*, 1990) or, when resection was performed in practically all patients with 'absolutely normal' CT scans: 86% (Aass *et al.*, 1991). Large European studies reported 31% (Dearnaley *et al.*, 1991) and 20% (Mead *et al.*, 1992), reflecting the policy to resect CT-detectable residual masses only if these exceed an arbitrarily chosen size. Further, subsets of patients have been defined (Donohue *et al.*, 1987; Fosså *et al.*, 1992), for whom the mortality and morbidity of resection may not be balanced by the small risk of leaving tumour unresected.

Our analysis of prognostic factors for relapse after resection showed (Table III) that significant prechemotherapy factors were the size (> 3 cm) and number of lung metastases. Although the cut off point for the prechemotherapy number of lung metastases at ⩾ 20 is applied rather sharply in clinical practice, it is obvious that the change in prognosis has a more gradual course; we found that a more accurate categorisation of the number of lung metastases is in three groups (⩽ 3, 4–19, ⩾ 20). Also, the initial serum value of the tumour marker HCG (⩾ 10,000 IU $l^{-1}$) has major prognostic impact. These factors were also found in other studies to predict relapse (Peckham, 1988; Stoter *et al.*, 1989; Stoter *et al.*, 1990; Bajorin *et al.*, 1991; Mead *et al.*, 1992), or to predict a complete clinical response after initial chemotherapy (Stoter *et al.*, 1990; Bajorin *et al.*, 1991). Post-chemotherapy adverse prognostic factors were the size (> 1 cm) of lung metastases, or the presence of any residual lung metastasis without detectable residual abdominal lymph metastases. Thus, the most important factors after chemotherapy and before resection, were the level of HCG and the extent of lung metastases.

The influence of the resection factors (completeness and histology) was studied in detail. Incompletely resected patients had a poor prognosis (5y-RF%: 50%), as was found in other studies (Tait *et al.*, 1984; Harding *et al.*, 1989; Jansen *et al.*, 1991). The size of retroperitoneal and lung metastases was significantly correlated with the completeness of resection. However, the adverse prognosis of incompletely resected patients was not explained by these factors, nor the prechemotherapy HCG level, nor the centre where the patient was resected. Thus, the patient had a poorer prognosis if the surgeon was unable to perform a complete resection, independent of other potential prognostic factors. An explanation for this finding might be that intrinsic tumour characteristics, such as chemosensitivity (Jansen *et al.*, 1991) or grade of malignancy of distinct tumour cell populations, are different in patients who could not be resected completely. This explanation is supported by the observation that of the five relapses in incompletely resected patients only one was definitely in the resection area.

The histology at resection was not related to relapse in our patients, similar to one other report (Harding *et al.*, 1989), but in contrast with the observations in several other studies (Tait *et al.*, 1984; Geller *et al.*, 1989; Jansen *et al.*, 1991; Mead *et al.*, 1992). The observation in our study may be explained by lack of power to detect an existing difference due to the relatively low number of relapses. A more interesting explanation is that the additional treatment after resection has been more effective than in other studies in controlling remaining microscopic disease, since a salvage chemotherapy regimen was used rather than two further cycles of the initial chemotherapy in ten of the 16 patients with viable cancer cells resected. The other six patients received two further cycles of the initial regimen ($n = 4$), radiation therapy after eight courses of chemotherapy before resection ($n = 1$), or no further treatment of a mesenchymal tumour ($n = 1$). This observation supports the recommendation to change the chemotherapy regimen after resection (Toner *et al.*, 1990).

The question rises whether incompletely resected patients might also benefit from a salvage regimen immediately after resection, even when no viable cancer cells are found in the resected material. The following observation suggests that benefit of additional chemotherapy might be obtained in these patients: six of the eight incompletely resected patients had no residual malignancy diagnosed (one necrosis, five mature teratoma). Five of these did not receive any additional chemotherapy after resection, and four relapsed. The other three patients received additional chemotherapy (one mature teratoma and two viable cancer cells resected) and only one relapsed, after 123 months.

According to our simple prognostic model (Figure 4), a poor prognosis is expected in patients with prechemotherapy HCG values over 10,000 IU $l^{-1}$ or an incomplete resection. The poor progrnosis of patients with a high prechemotherapy level of HCG is already being recognised by a number of treatment groups (Stoter *et al.*, 1990; Bajorin *et al.*, 1991; Mead *et al.*, 1992), and these patients are candidates to receive more intensive induction chemotherapy. Improvement of the prognosis of incompletely resected patients might be obtained by the administration of salvage chemotherapy after resection, although further research has to confirm this. The use of a salvage regimen after resection rather than two further cycles of the same chemotherapy is also subject to further investigation as well as more detailed recommendations for the selection of patients who would benefit from surgical resection.

We would like to thank Jo Hermans, PhD, Department of Medical Statistics, University of Leiden, for statistical support.

### References

AASS, N., KLEPP, O., CAVILLIN-STÅHL, E., DAHL, O., WICKLUND, H., UNSGAARD, B., BALTETORP, L., AHLSTRÖM, S. & FOSSÅ, S.D. (1991). Prognostic factors in unselected patients with nonseminomatous metastatic testicular cancer: a multicenter experience. *J. Clin. Oncol.*, **9**, 818–826.

BAJORIN, D.F., GELLER, N.L. & BOSL, G.J. (1991). Assessment of risk in metastatic testis carcinoma: impact on treatment. *Urol. Int.*, **46**, 298–303.

COX, D.R. (1972). Regression models and life tables (with discussion). *J. Roy. Stat. Soc. Ser. B.*, **34**, 187–220.

DEARNALEY, D.P., HORWICH, A., A'HERN, R., NICHOLLS, J., JAY, G., HENDRY, W.F. & PECKHAM, M.J. (1991). Combination chemotherapy with bleomycin, etoposide and cisplatin (BEP) for metastatic testicular teratoma: long-term follow-up. *Eur. J. Cancer*, **27**, 684–691.

DONOHUE, J.P. & ROWLAND, R.G. (1984). The role of surgery in advanced testicular cancer. *Cancer*, **54**, 2716–2721.

DONOHUE, J.P., ROWLAND, R.G., KOPECKY, K., STEIDLE, C.P., GEIER, G., NEY, K.G., EINHORN, L., WILLIAMS, S. & LOEHRER, P. (1987). Correlation of computerized tomographic changes and histological findings in 80 patients having radical retroperitoneal lymph node dissection after chemotherapy for testis cancer. *J. Urol.*, **137**, 1176–1179.

EINHORN, L.H. (1990). Treatment of testicular cancer: a new and improved model. *J. Clin. Oncol.*, **8**, 1777–1781.

FOSSÅ, S.D., QVIST, H., STENWIG, A.E., LIEN, H.H., OUS, S. & GIERCKSKY, K.E. (1992). Is postchemotherapy retroperitoneal surgery necessary in patients with nonseminomatous testicular cancer and minimal residual tumor masses? *J. Clin. Oncol.*, **10**, 569–573.

GELLER, N.L., BOSL, G.J. & CHAN, E.Y.W. (1989). Prognostic factors for relapse after complete response in patients with metastatic germ cell tumors. *Cancer*, **63**, 440–445.

HARDING, M.J., BROWN, I.L., MACPHERSON, S.G., TURNER, M.A. & KAYE, S.B. (1989). Excision of residual masses after platinum based chemotherapy for non-seminomatous germ cell tumours. *Eur. J. Cancer Clin. Oncol.*, **25**, 1689–1694.

JANSEN, R.L.H., SYLVESTER, R., SLEYFER, D.T., TEN BOKKEL HUININK, W.W., KAYE, S.B., JONES, W.G., KEIZER, H.J., VAN OOSTEROM, A.T., MEYER, S., VENDRIK, C.P.J., DE PAUW, M. & STOTER, G. for the EORTC GU Group (1991). Long-term follow-up of non-seminomatous testicular cancer patients with mature teratoma or carcinoma at postchemotherapy surgery. *Eur. J. Cancer*, **27**, 695–698.

KAPLAN, E.L. & MEIER, P. (1958). Nonparametric estimates from incomplete observations. *J. Am. Stat. Assoc.*, **53**, 457–481.

LEVITT, M.D., REYNOLDS, P.M., SHEINER, H.J. & BYRNE, M.J. (1985). Non-seminomatous germ cell testicular tumours: residual masses after chemotherapy. *Br. J. Surg.*, **72**, 19–22.

MANTEL, N. (1966). Evaluation of survival data and two new rank order statistics arising in its consideration. *Cancer Chemother. Rep.*, **50**, 163–170.

MEAD, G.M., STENNING, S.P., PARKINSON, M.C., HORWICH, A., FOSSÅ, S.D., WILKINSON, P.M., KAYE, S.B., NEWLANDS, E.S. & COOK, P.A. for the Medical Research Council Testicular Tumour Working Party (1992). The second medical research council study of prognostic factors in nonseminomatous germ cell tumors. *J. Clin. Oncol.*, **10**, 85–94.

MULDERS, P.F.A., OOSTERHOF, G.O.N., BOETES, C., DE MULDER, P.H.M., THEEUWES, A.G.M., & DEBRUYNE, F.M.J. (1990). The importance of prognostic factors in the individual treatment of patients with disseminated germ cell tumours. *Br. J. Urol.*, **66**, 425–429.

NEWLANDS, E.S. & REYNOLDS, K.W. (1989). The role of surgery in metastatic testicular germ cell tumours (GCT). *Br. J. Cancer.*, **59**, 837–839.

PECKHAM, M. (1988). Testicular cancer. *Rev. Oncol.*, **1**, 439–453.

PUGH, R.C.B. (1976). Testicular tumours, introduction. In *Pathology of the Testis*, Pugh, R.C.B. *et al.* (eds) pp. 139–162. Blackwell Scientific Publishers: Oxford.

STOTER, G., KOOPMAN, A., VENDRIK, C.P.J., STRUYVENBERG, A., SLEYFER, D.TH., WILLEMSE, P.H.B., SCHRAFFORDT KOOPS, H., VAN OOSTEROM, A.T., TEN BOKKEL HUININK, W.W. & PINEDO, H.M. (1989). Ten-year survival and late sequelae in testicular cancer patients treated with cisplatin, vinblastine, and bleomycin. *J. Clin. Oncol.*, **7**, 1099–1104.

STOTER, G., BOSL, G.J., DROZ, J.P., FOSSÅ, S.D., FREEDMAN, L.S., GELLER, N.L., HORWICH, A., JONES, W.G., KAYE, S.B., MEAD, G.M., OOSTEROM, R., RODENBURG, C.J., SCHEULEN, M.E., STENNING, S., SYLVESTER, R. & VOGELZANG, N.J. (1990). Prognostic factors in metastatic germ cell tumors. In *EORTC Genitourinary Group Monograph 7: Prostate Cancer and Testicular Cancer*, Newling, D.W.W., Jones, W.G. (eds) pp. 313–319. Wiley-Liss, Inc.: New York.

TAIT, D., PECKHAM, M.J., HENDRY, W.F. & GOLDSTRAW, P. (1984). Post-chemotherapy surgery in advanced non-seminomatous germ-cell testicular tumours: the significance of histology with particular reference to differentiated (mature) teratoma. *Br. J. Cancer*, **50**, 601–609.

TONER, G.C., PANICEK, D.M., HEELAN, R.T., GELLER, N.L., LIN, S.-Y. BAJORIN, D., MOTZER, R.J., SCHER, H.I., HERR, H.W., MORSE, M.J., FAIR, W.R., SOGANI, P.C., WHITMORE, Jr, W.F., MCCORMACK, P.M., BAINS, M.S., MARTINI, N. & BOSL, G.J. (1990). Adjunctive surgery after chemotherapy for nonseminomatous germ cell tumors: recommendations for patient selection. *J. Clin. Oncol.*, **8**, 1963–1694.